# Raise up of Scopolamine in Hairy Roots Via *Agrobacterium rhizogenes* ATCC15834 as Compared with Untransformed Roots in *Atropa komarovii*

**DOI:** 10.22037/ijpr.2019.13550.11710

**Published:** 2020

**Authors:** Ofelia Banihashemi, Ramazan-Ali Khavari-Nejad, Narguess Yassa, Farzaneh Najafi

**Affiliations:** a *Department of Biology, Science and Research Branch, Islamic Azad University, Tehran, Iran. *; b *Department of Pharmacognosy, Faculty of Pharmacy, Tehran University of Medicinal Sciences, Tehran, Iran. *; c *Department of Plant Science, Faculty of Biological Science, Kharazmi University Tehran, Iran.*

**Keywords:** Atropa komarovii, rol B, Secondary metabolite, Transformation, Tropane alkaloids

## Abstract

*Atropa komarovii* generates tropane alkaloids and three other compounds such as hyoscyamine. *Racemate atropine* and *scopolamine* (hyoscine) are the main alkaloids with anticholinergic, antispasmodic, and sedative agents. A proficient convention has been reported for the formation of transgenic *Atropa komarovii* by the use of *Agrobacterium rhizogenes.* Root culture, by utilizing leaves explants was contaminated by *Agrobacterium rhizogenes* ATCC 15834, a strain with the paired vector. The hairy roots after contamination for three weeks were specifically shaped from the cut edges of the leaves. The PCR intensification demonstrated that *rol* B genes of *Ri* plasmid of *Agrobacterium rhizogenes* were coordinated and communicated into the genome of the changed hairy roots. Examination of HPLC revealed that hairy roots can produce scopolamine and hyoscyamine and it was appeared that scopolamine content was essentially expanded in changed roots and hyoscyamine was extremely expanded in non-transgenic roots. According to the results, it was perceived that the scopolamine content in hairy roots was raised significantly compared to the control roots. It was evidenced that hairy roots gather a great number of metabolites that have a commercial significance. Thus, later on we can enhance efficiency for example by building up the biosynthetic route overexpression of gene codifying enzymes in the metabolic route for expanding valuable secondary metabolites in the plant cures.

## Introduction

*Atropa komarovii* (Blin &Shal) is a kind of lasting herbaceous plant, and the most critical source of pharmaceutical tropane alkaloids in the solanaceae family in commercial affairs. Nowadays, alkaloids are one of the countless extricated materials with biological activity. Many attempts have been done to build up these alkaloids in biotechnological methods via *Agrobacterium rhizogenes* that is a gram negative bacterium (1). The reason for biosynthetic unsustainability in plant cell culture is innate heterogeneity of cells, ecological anxiety and the absence of tissue separation absolutely (2). Hairy roots develop quickly with stable and relatively high substance in secondary metabolites, inducted by genetic transformation through *A.rhizogenes* that were effectively employed for the generation of secondary metabolites from pharmaceutical or fragrant plants (3). Distinctive plant tissues from most dicotyledonous and monocotyledonous species have exhibited the ability to be tainted and genetically changed by Ri T-DNA of *Agrobacterium rhizogenes*, bringing about development of hairy roots (4). T-DNA will be incorporated into plant chromosomes and express genes to combine opine material and oncogene coding the development hormone of auxin and cytokinin (5). It was demonstrated that for improvement of the hairy root ailment, the attendance of four *rol*-genes, for example, *rol* A, *rol* B, *rol* C, and *rol* D are vital, by the utilization of insertion and elimination mutagenesis (6). A few investigations uncovered a fascinating capacity of the *rol* genes to fortify secondary metabolite creation in transgenic plant tissues and as the changed root cultures have presented extra advantages, for instance, quick development, consistency, hereditary solidness, and high biosynthetic ability (7). Hairy root culture is a type of plant tissue culture that is used to scrutiny metabolic procedures of plant, generation of recombinant proteins, plant genetic designing, phytoremediation, artificial seed creation, biofortification, and biopharmaceuticals (8). Tropane alkaloids are incorporated in the youthful root cells and after that shipped to the aeronautical parts of the plant, but cell cultures of various solanaceae species have shown low tropane alkaloid generation, mainly because of the absence of separation (9). It seems that no examination has been accounted for about evaluation of the hairy roots induction, development and tropane alkaloid creation in *Atropa komarovii *hairy roots with these lines. This examination was directed to decide the best situation for hairy root arrangement and for raising tropane alkaloids generation.

## Experimental

*Plant material and culture condition*

The seeds of *Atropa komarovii* were gathered from the Gozluge urben on Ali-abad street, Goletan region located at 3000 m height. Latency of the seeds was broken by scratch. The seeds of *Atropa komarovii* were surface disinfected with 70% (v/v) sodium hypochlorite solution for 15 min took after by three rinse with refined water. They were cultured on solid hormone-free Murashige and skoog media (MS) including 30 gL^-1^ sucrose for one week in a growth chamber at 26±2 ºC and 16/8 h (light/dark) photoperiod with a photon transition thickness of 60 µ moL/ms2.

*Bacterial strains*

*Agrobacterium rhizogenes* strain ATCC15834 catered by Zist Fanavaran Organization, Tehran, Iran, was raised on Luria – Bertani solid medium (LB: contains: 5 g/L) Bacto-yest extract, 10 g/L Bacto-trypton and 10 g/L NaCl, conform to pH 7.0) as needed. Before infection, single clone was developed for 24 h in LB fluid medium containing rifampicin (50 mgr L^-1^) at 28 ºC on revolving shaker at 200 rpm in the dark, at that point of the optical thickness was kept at 600 nm (OD = 0.7) by means of spectrophotometer.

*Establishment of hairy root cultures*

The leaves of *Atropa komarovii* were taken from micro-propagated shoots raised under vitro condition at age of three weeks. The extracted leaves were injured with surgical blade and drenched into *A.rhizogenes* ATCC 15834 for 20 min at that point smeared dry on sterile strainer paper, and hatched on MS solid media at 25 ºC in the dark. After 2 days of co-development, immunized leaves were exchanged to a MS medium without hormone containing 500 mg L^-1^ cefotaxime. Cefotaxime concentration was then divided every three weeks from 500 mg L^-1^ to 250 mg L^1^. Various hairy roots were initiated from twisted locales of leaf explants inside 2 weeks after immunization.

*Hairy root growth*

The hairy roots were isolated from leaves and sub-cultured on agar solidfied MS medium at regular intervals under standard white fluorescent tubes and 16 h light/8 h dark photoperiod and 26 ºC. The hairy roots were gathered after 21 days to test the dry and fresh weight.

*Fresh weight (FW) and dry weight (DW)*

Following three weeks, the hairy root and control root were prompted, at that point we drag them out of the medium and cleaned them with refined water, and weighted (FW) them after elimination of the water with strainer paper. The dry weight was measured in the point when the fresh roots were dried at 60 ºC in an electric oven. This was triplicated during for successive weeks on each sample.

*Extraction of DNA*

The whole DNA was secluded from hairy roots and ordinary roots (control) of Genomic DNA were extracted from hairy roots and ordinary roots (control) of *Atropa komarovii* by the cetyl – trimethyl ammonium bromide (CTAB) strategy (10). Fresh hairy root tissues 0.4g were reaped, frozen in fluid nitrogen, and grounded into fine powder. The frozen powder was moved into two mL micro-centrifuge tubes and homogenized in 0.8 mL of CTAB extraction Buffer (2% CTAB, 100 mM Tris-HCl (pH 8), 20 mM, EDTA,1.4 NaCl, 0.1 mL protein as K) and hatched at 60 °C for 1 h and it included 0.8 mL chloroform/isoamylalcohol (24:1) arrangement. The supernatant was transferred to another tube and 0.06 mL isopropanol was included and centrifuged for 15 min at 1400 g. The plate was dried by leaving tube open for 25 min and afterward reacted in 50 µL TE (10 Mm Tris – HCl, PH 7, and 1mM EDTA, P^H^ 8).

*PCR analysis for rol B*

The whole genomic DNA was separated in light of CTAB technique from each of the hairy root as well as the control roots (non-changed) Their activity was completed using *rol* B (500bp) gene particular preliminaries forwar5’ACCGATCCCAAATTGCTATTCCCCACGA 3’ and inverse 5’ AATGGCTTCTTTCATTCGGTTTACTGCAGC 3’ respectively according to Hashimoto *et al. *PCR response was completed in 20 µL containing H_2_O 14.7 mL, PCR buffer 20 μL, dNTP 0.5 μL, 50 ng of genomic DNA, 1 u of Taq DNA polymerase, 10 p moles preliminaries, and MgCl_2_ 2 Mm. Amplification cycle included introductory denaturation at 94 ºC for 5 min , trailed by 30 cycles of 1 min denaturation at 94 ºC, 1 min annealing at 57 ºC , 1 min expansion for 72 ºC and last augmentation at 72 ºC for 10 min in termocycler. 

*Tropane alkaloid extraction*

The upper parts, for example, the control roots, untransformed roots, roots and leaves of *Atropa komarovii* plantlet were finely powdered in a mill, and 1gr of each sample was macerated in hexane. The dissolvable was evacuated under diminished weight at low temperature. The remainder was re-removed using methanol. The solvents were vaporized under the reduced pressure.The subsequent extract was broken down by a HPLC method utilizing with slight modification (12). 

*HPLC analysis of Tropane alkaloids*

The HPLC examinations of the alkaloids were done by slight adjustment. Scopolamine and Hyoscyamine were broken down through high-performance liquid chromatography. The HPLC framework had an ultraviolet– visible detector and a C8 reverse-phase column. The versatile phase was H_3_PO_4_ 50 Mm (pH 2.9), acetonitril: 85:15 (v/v). The stream speed was 1 mL for every moment (13) The identification wavelength was 254 nm. The sample solution of infusion was 20 µL each time The standard sample and hyoscyamine scopolamine were set up in methanol and diluted into 10, 20, 40, and 60 ppm and were acquired through serial dilution of the stock solution with methanol. The calibration diagrams for the standard samples were built through plotting the pinnacle territory of the alkaloids against their concentrations. Linear calibration graphs were gotten with great connection for standard arrangements.

*Statistical analysis*

Each part of data was the mean of three replicates. Statistical analysis was performed utilizing SPSS software (version 22). We used t-test and the univariate strategy at *P *< 0.05.

**Figure 1:**
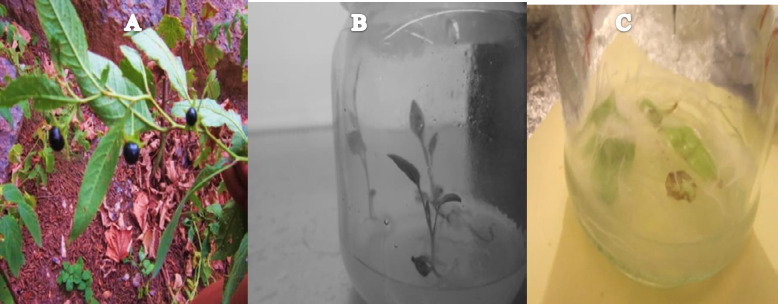
**A.** Fruit of *Atropa komarovii *.B Seed development of *Atropa komarovii* on MS hormone-free medium. C: Hairy root appeared at the cut edge of a leaf explants

**Figure 2:**
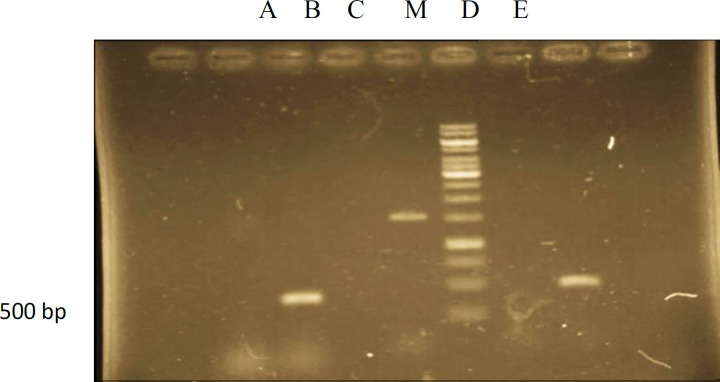
PCR analysis of hairy root culture of *Atropa komarovii* transformed *Agrobacterium rhizogenes* ATCC 15834. lane M –Marker (1kbp): lane A- genomic DNA of hairy root culture showing amplified fragment of *rol*B (500bp): lane B- genomic DNA from normal root culture (negative control): lane C- PCR(positive control): lane D - PCR (negative control): lane E- genomic DNA of hairy root culture showing amplified fragment of *rol*B (500bp).

**Figure 3:**
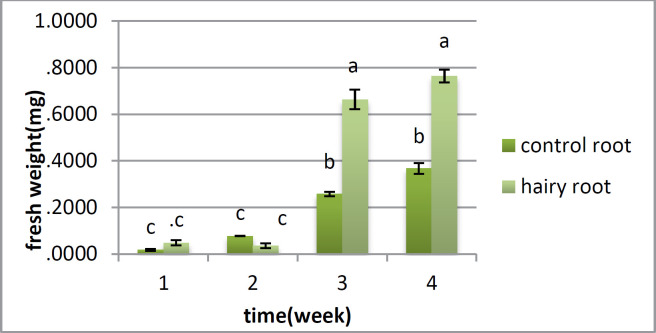
Changes of fresh weight in hairy root and control during four weeks in *Atropa komarovii* .Different letters on bars refers to significant difference (*P *<0.05).

**Figure 4.**
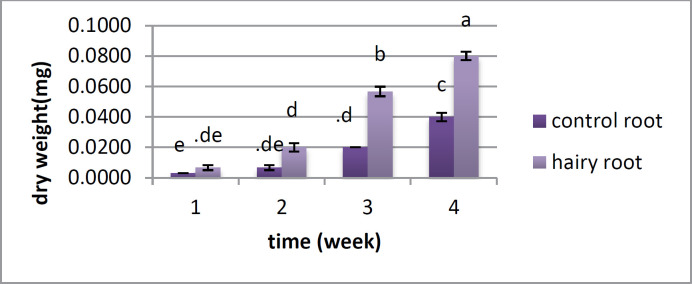
Changes of dry weight in hairy root and control during four weeks in *Atropa komarovii*. Different letters on bars refers to significant difference (*P*<0.05)

**Figure 5:**
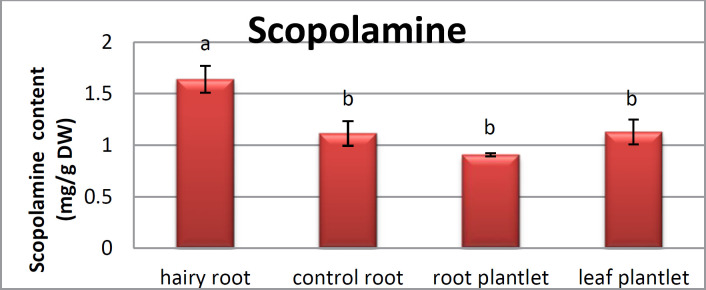
Analyses for scopolamine contents in hairy root,control roots, root and leaf plantlet of *Atropa komarovii*

**Figure 6:**
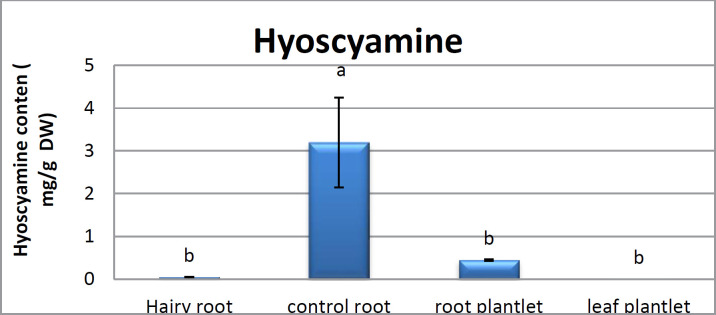
Analyses for hyoscyamine contents in hairy root, control roots, root and leaf plantlet of *Atropa komarovii*

**Figure 7:**
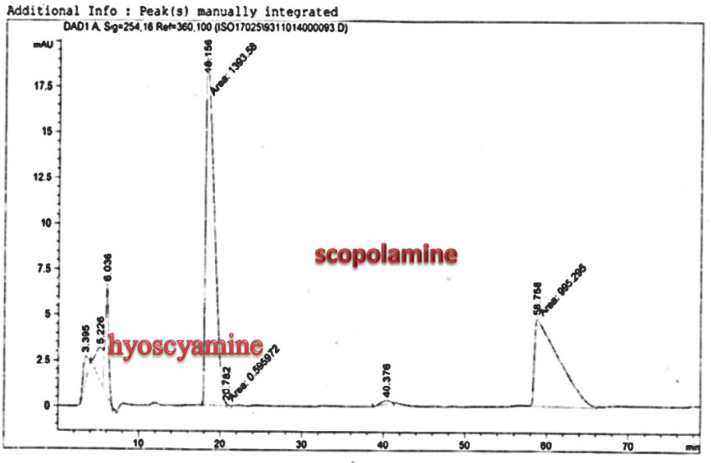
Analysis of Scopolamine and hyoscyamine standard HPLC chromatogram

**Figure 8.**
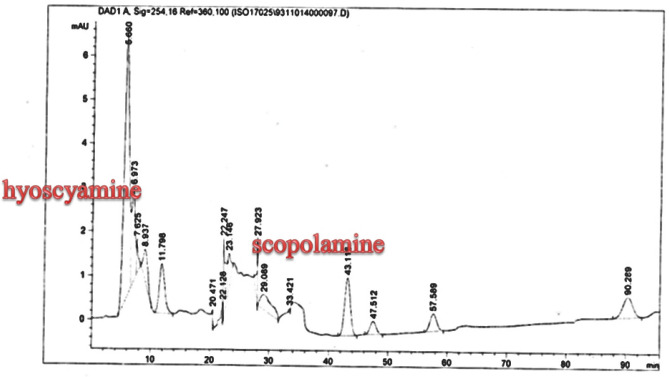
Analysis of Scopolamine and hyoscyamine HPLC chromatogram of *Atropa komarovii* hairy roots culture

**Figure 9.**
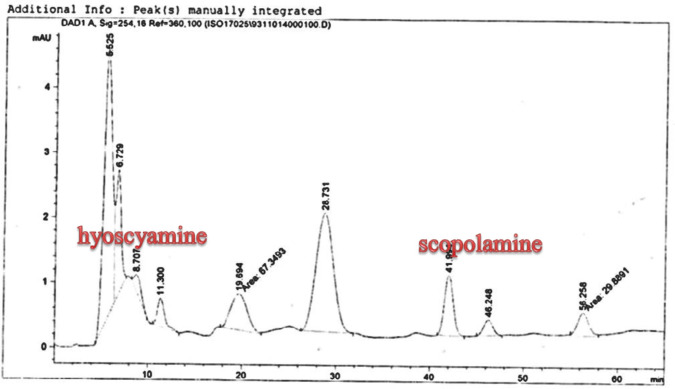
Analysis of Scopolamine and hyoscyamine HPLC of *Atropa komarovii* control roots culture

**Figure 10:**
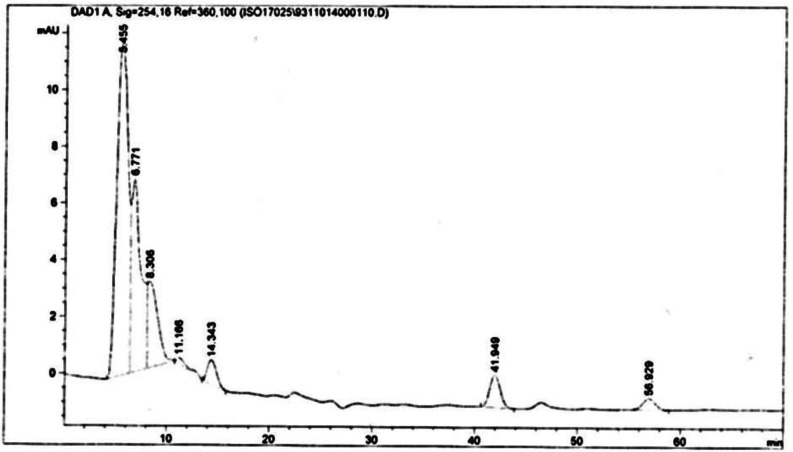
Analysis of Scopolamnine and hyoscyamine HPLC of *Atropa komarovii* leaves culture

**Figure 11:**
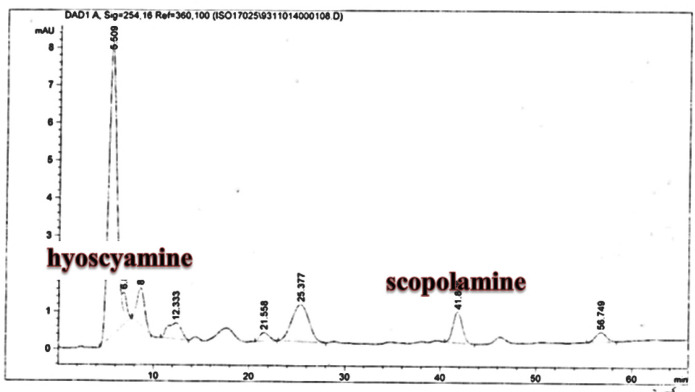
Analysis of Scopolamine and hyoscyamine HPLC of *Atropa komarovii * root culture

## Results and Discussion

*Induction of the hairy roots*

The fresh leaves of *Atropa komarovii* plantlet with 6 or 7 leaves demonstrated high affectability to ATCC15834 (Figure 1). The high change recurrence was almost seen with 60% from leaf explants vaccinated by means of *A.rhizogenes* and other *Atropa* species such as *belladonna* and baetica have been genetically modified by *Agrobacterium rhizogenes *(14) The hairy roots and control roots were extracted from explants and exchanged to new MS medium with no auxin, and the hairy roots, fit for blending endogenous auxin, in this manner did not require exogenous auxin. After one month, some control roots were not survived but rather changed roots achieved greatest biomass, which had lots of horizontal expanding, thick, negative geotropism, and stretched quickly (Figure 1, A,B,C). Tissue culture, joined with genetic designing particularly changed innovation that caused fulfillment and opened new ways for high volume generation of pharmaceutical substances (15). This examination demonstrated an effective technique for prompting hairy roots in *Atropa komarovii*. The outcomes introduced in this reveal that the utilization of *A.rhizogenes* may be a fruitful way to deal with hairy root infusion (16). In the contamination method, the initial phase is the host/pathogen interface where a few components commit to the foundation of this connection. Phenolic compounds and sugars secreted from the plants, according to plant genotype, thus created distinctive reactions (17). *Agrobacterium rhizogenes* intervened genetic change in plants is a settled auxin: cytokinin proportion in the plant cell. Since different plants can demonstrate discriminative susceptibility to a given *Agrobacterium rhizogenes* strain. *A. rhizogenes* that was harboring double vector was developed with leaves of *A.komarovii* approximately following three week of incubation on MS basal medium. The roots rose straightforwardly from the site of contamination on leaf discs, at the injured locals (18).

*PCR analysis of rol B gene*

To affirm the joining of T-DNA from the *A.rhizogenes* into the hairy root genomic DNA, DNA from hairy roots were subjected to PCR examination. PCR was utilized to show that the T-DNA from the Ri plasmid of *A.rhizogenes* was available in *A.komarovii* genome and *rol* B is one gene of the TL-DNA (T-DNA left arm) of Ri plasmid in *A.rhizogenes*. *A.komarovii* changed with the *rol* B gene of *A. rhizogenes* during the ceaseless sub-culturing. In this investigation, by utilizing DNAs from the hairy roots as template and non-transform roots as a control, PCR products opened up with *rol* B forward and switch primers could be identified It was exhibited that every single changed root demonstrated the 500 bp *rol* B gene and there was no *rol* B gene found in control roots (Figure 2). The biochemical and molecular characterization of the T-DNA genes from *A.rhizogenes* is not completely comprehended and in a few investigations, proposing that new meristem development and consequent separation of changed plant cells might be directed through complex molecular systems (19). To affirm the joining of T-DNA from the *A.rhizogenes* into the hairy root genomic DNA, DNA from hairy roots were subjected to PCR examination. PCR was utilized to show that the T-DNA from the Ri plasmid of *A.rhizogenes* was available in *A.komarovii* genome and *rol* B is one gene of the TL-DNA (T-DNA left arm) of Ri plasmid in *A.rhizogenes*. *A.komarovii* changed with the *rol* B gene of *A. rhizogenes* during the ceaseless sub-culturing. In this investigation, by using DNAs from hairy roots as template and non transform roots as a control, PCR products opened up with *rol* B forward and the switch primers could be identified (20). We performed DNA investigation, be aware of that end goal to affirm the hairy roots change. The TL district in plasmid T-DNA agropine sort strain *Agrobacterium rhizogenes* ATCC15834 contain 18 operon perusing outlines including a few loci (root loci) (and the impact of TR and TL locales of *A. rhizogenes* on development hairy roots (21). Numerous different were additionally brought up that the *rol* genes are actuated hairy roots and *rol* B is an effective inducer of secondary metabolism in transgenic plants (22). In this examination increment in the quantity of hairy roots the branches was practically logarithmic and we revealed high scopolamine creation by hairy root of *A.komarovii* changed with the *rol* B gene of *A. rhizogenes* amid of persistent sub-culturing. Numerous different reports additionally brought up that the *rol* genes are prompted hairy roots and *rol* B is a capable inducer secondary metabolism in transgenic plants (23). Hairy roots quickly developed with stable and relatively high substance in secondary metabolites and actuated by genetic change of *A.rhizogenes*, were effectively utilized for the creation of secondary metabolites from medicinal or fragrant plants and genetic changed root cultures may deliver the secondary metabolites like that of the intact plants (24). 

*Analysis for fresh weight and dry weight*

There is no distinction between the hairy root and control root in fresh weight in the first and second weeks; however the huge change was found in the third and fourth weeks, when the fresh weight of the roots was higher than that of the control roots (Figure 3). Dry weight showed a critical distinction between the two samples from the first week, and this distinction was observed over a month. In the first week, there was no huge distinction between them; however there was a contrast between the hairy roots and control roots from the second to the fifth week (Figure 4).

*Scopolamine and hyoscyamine analysis*

The hairy roots quickly developed with stable and relatively high substance in secondary metabolites, actuated by genetic change of *A.rhizogenes*, were effectively used for the creation of secondary metabolites from medicinal or fragrant plants and the genetic changed root cultures may deliver the secondary metabolites like that of the intact plants (25). The substance of scopolamine and hyoscyamine were recognized in the hairy roots, non-transformed roots, roots, and leaves of the plantlet and both of these tropane alkaloids could be identified in all samples The maintenance time of the scopolamine substance of the hairy root culture was contrasted with the standard scopolamine and hyoscyamine (Sigma) In the HPLC profile, the RT value indicated the same points for both the standard and the concentration of the hairy root culture (Figure7). The outcomes concerning the primary tropane alkaloids (scopolamine and hyoscyamine) from *Atropa komarovii* plant tissue with hairy roots demonstrate higher measure of scopolamine than hyoscyamine in hairy roots (Figure 5). From the outcomes concerning the fundamental oppositely, we found that the greatest measure of hyoscyamine was available in the control roots and on the other hand, scopolamine was the highest content compared to other treatments such as a leaves and roots of *Atropa komarovii* plantlet (Figure 5 and 6). Pathway-designing hairy root cultures are not just the perfect arrangement of recognize genetic capacities and in the past reports, transgenic hairy root cultures of *Atropa belladonna* were stablished by over expressing different genes with the biosynthetic pathway of tropane alkaloids (26). This result indicates that concurrence with information on scopolamine aggregation in hairy roots of *Atropa belladonna* and shows that there are many elements participate in scale-up scopolamine in tissue culture revealing that expansion of scopolamine content in hairy roots of *A. belladonna* by means of bioreactor (27). We observed a considerable increment of scopolamine content in hairy roots and hyoscyamine was increased in nontransformed roots (Figure 6, 8, 9, 10 and 11). Biosynthesis of secondary metabolite in changed roots is genetically controlled. In many reports in other genera of solanaceae transgene, invigorated scopolamine creation and its scopolamine synthesis appears to be metabolic regulation (7). The nutritive factors on the production of two tropane alkaloids, scopolamine, and hyoscyamine in *Atropa belladonna* hairy roots, the reserchers observed that the amount of hyoscyamine increased in hairy roots compared to plant leaves and roots. This results opposed to our studies and scopolamine content in the hairy roots were 10 times higher than in organs of intact plants (Figure 6). Chashemi *et al*. indicated that synthesis of hyoscyamine and scopolamine in *Atropa belladonna* hairy roots increased than control roots and the hyoscyomaine is more than scopolamine in hairy roots and it was against of our reports (22). Tropane alkaloid distribution in *Atropa baetica* plants and they said hyoscyamine was more abundant, with the highest concentration in the main root followed by leaves and then scopolamine present in highest concentration in the main root (26). In our researches, hyoscyamine in control root was higher than root plantlet and hairy roots and scopolamine were obsereved in all the samples although it has the highest amounts in hairy roots. Banerjee *et al*. studied the expression tropane alkaloids in hairy root culture of *Atropa acuminate* scopolamine that was higher in hairy root than hyoscyamine and the major tropane alkaloids was scopolamine and hyoscyamine in *Atropa acuminate* (24).
